# A DFT Study of Pyrrole-Isoxazole Derivatives as Chemosensors for Fluoride Anion

**DOI:** 10.3390/ijms130910986

**Published:** 2012-09-05

**Authors:** Ruifa Jin, Weidong Sun, Shanshan Tang

**Affiliations:** 1 College of Chemistry and Chemical Engineering, Chifeng University, Chifeng 024000, China; E-Mail: Cfxyhxx@163.com; 2 College of Resource and Environmental, Jilin Agricultural University, Changchun 130118, China; E-Mail: shanshantang@yahoo.cn

**Keywords:** pyrrole-isoxazole derivatives, chemosensor, BSSE (counterpoise) correction, intramolecular charge transfer, atoms in molecules

## Abstract

The interactions between chemosensors, 3-amino-5-(4,5,6,7-tetrahydro-1*H*-indol-2-yl)isoxazole-4-carboxamide (**AIC**) derivatives, and different anions (F^−^ Cl^−^, Br^−^, AcO^−^, and H_2_PO_4_^−^) have been theoretically investigated using DFT approaches. It turned out that the unique selectivity of **AIC** derivatives for F^−^ is ascribed to their ability of deprotonating the host sensors. Frontier molecular orbital (FMO) analyses have shown that the vertical electronic transitions of absorption and emission for the sensing signals are characterized as intramolecular charge transfer (ICT). The study of substituent effects suggests that all the substituted derivatives are expected to be promising candidates for fluoride chemosensors both in UV-vis and fluorescence spectra except for derivative with benzo[*d*]thieno[3,2-*b*]thiophene fragment that can serve as ratiometric fluorescent fluoride chemosensor only.

## 1. Introduction

The design and synthesis of receptors capable of binding and sensing anions selectively have received significant attention in recent years because anions play an important role in a wide range of biological, environmental, and chemical processes [1–6]. Especially, colorimetric and ratiometric chemosensors appear to be particularly attractive due to their simplicity, high sensitivity, and high selectivity [7,8]. For quantitative analyses, ratiometric chemosensors have the significant advantage of their dual emission system, which can minimize measurement errors caused by fluctuations of light scattering as well as reagent concentration [9]. Colorimetric chemosensors provide an immediate qualitative signal, which allows direct naked-eye detection of anions because of a specific color change of solution upon anion complexation [10,11]. Among the range of biologically important anions, fluoride, the smallest anion, has unique chemical properties. It is of particular interest to recognize and detect it owing to its essential role in a broad range of biological, medical, and chemical processes of osteoporosis, fluorination of drinking water supplies, or even in chemical and nuclear warfare agents [12–14]. However, the chemosensors for F^−^ are rather few, and there is a need for good anion sensors with an improved specific response, in particular, with a selectivity for F^−^ in the presence of other anions. Furthermore, it is necessary to understand the unique host-guest interaction of the sensor with F^−^ and other anions. Different signaling mechanisms have been suggested for F^−^, such as photoinduced electron transfer (PET) [15], excited state proton transfer (ESPT) [16], intramolecular charge transfer (ICT) [17,18], excimer and exciplex formation [19], and metal-ligand charge transfer (MLCT) [20], *etc*.

Recently, a fluoride chemosensor made of a derivative of pyrrole-isoxazole, 3-amino-5-(4,5,6, 7-tetrahydro-1*H*-indol-2-yl)isoxazole-4-carboxamide (**AIC**, Scheme I), has been reported [21]. **AIC** shows both changes in its UV-vis absorption and fluorescence emission spectra upon the addition of F^−^, resulting in a higher selectivity for fluoride detection compared to other anions in CH_3_CN. Interactions of **AIC** with F^−^ cause a red-shift in UV-vis absorption and a Stokes shift in fluorescence emission due to the deprotonation of the active pyrrole-NH moiety of **AIC** by F^−^. To the best of our knowledge, neither calculations of the host-guest interactions with the basis set superposition error (BSSE) corrections nor sophisticated level optimizations for the neutral, anion, and complexes forms of **AIC** derivatives in ground states (S_0_) and first excited singlet states (S_1_) have been reported so far.

Herein we report the investigation of both host-guest interaction and signaling properties from a theoretical point of view for this system. Further in-depth explanations for the experimental results have been discussed by the investigation of the optical and electronic properties of pyrrole-isoxazole derivatives. To investigate the substituent effect, several derivatives (**1**–**5**), as shown in Scheme I, have been designed by introducing functional groups to pyrrole-isoxazole to provide a demonstration for the rational design of new fluorescent and/or chromogenic chemosensors for fluoride anion.

## 2. Computational Details

All calculations were performed using Gaussian 09 code [22]. Optimizations were carried out without symmetry constraints. The geometries of **AIC** and **1**–**5** and their anions in S_0_ were optimized by using the hybrid B3LYP functional with 6-31+G(d,p) basis set. Furthermore, complexes consisting of **AIC** and **1**–**5** and *X*^−^ (*X* = F, Cl, Br, AcO, and H_2_PO_4_) were optimized at the same theoretical levels as above with the consideration of the BSSE correction using the counterpoise method [23]. The unphysical long-range asymptote of the exchange part of popular functionals B3LYP makes them unsuitable for calculations of excited states having a large charge-transfer (CT) component [24,25]. Therefore, the S_1_ structures for **AIC** and **1**–**5** and their anions were optimized using the long-range corrected version of B3LYP using the Coulomb-attenuating method CAM-B3LYP functional, which contains 65% long-range HF exchange [25,26]. These range-separated functionals rely on a growing fraction of exact exchange when the interelectronic distance increases, subsequently providing a more physically sound model for long-range phenomena. It has been shown that the CAM-B3LYP functional proved efficient in the determination of the charge-transfer transition [27–31]. The harmonic vibrational frequency calculations using the same methods as for the geometry optimizations were used to ascertain the presence of a local minimum. Absorption and fluorescent properties of **AIC** and **1**–**5** and their anions were predicted using the TD-B3LYP/6-31+G(d,p) and TD-BLYP/6-31+G(d,p) methods based on the S_0_ and S_1_ optimized geometries, respectively. To investigate the influence of solvents on the optical properties for the S_0_ and S_1_ states of the molecular systems in CH_3_CN (dielectric constant: 35.688) solvent, we performed the polarized continuum model (PCM) [32] calculations at the TD-DFT level.

To gain an additional insight into the bonding characteristics of the studied complexes consisting of **AIC** and **1**–**5** and *X*^−^ (*X* = F, Cl, Br, AcO, and H_2_PO_4_), we also used the atoms in molecules (AIM) [33,34] theory at the B3LYP/6-31+G(d,p) level. The charge distribution was performed using the natural bond orbital (NBO) approach, which has been proven to be very valuable in the study and characterization of hydrogen bonds and cooperative effects [35].

## 3. Results and Discussion

### 3.1. Host-Guest Interaction

In order to obtain an insight into the anion-sensing mechanism, the interactions between hosts and guests were investigated exploiting the density functional method. Table 1 presents the most important geometrical parameters with and without BSSE corrections for complexes **AIC**^−^·HF and **AIC**·*X*^−^ (*X* = Cl, Br, AcO, and H_2_PO_4_). The corresponding geometrical parameters of complexes for **1**–**5** are given in Table SI, Supporting Information. According to the suggested geometry cutoffs for D–H···A hydrogen bond definition, *i.e.* H···A distances <3.0 Å and D–H···A angles >110° [36,37], the interaction between pyrrole N and H for complex **AIC**^−^·HF and between Cl, Br, AcO, and H_2_PO_4_ and pyrrole-NH in **AIC**·*X*^−^ (*X* = Cl, Br, AcO, and H_2_PO_4_) can be identified as hydrogen bonds. Comparing the optimized geometries with and without BSSE correction, one may find that the deviations of the hydrogen bond length between pyrrole-N and H (*R*_N_·_H_) and the distance between H and F (*R*_H–F_) for complex **AIC**^−^·HF are 0.005 and 0.001 Å, respectively. The angles *θ*_N–H_·_F_ in complex **AIC**^−^·HF without BSSE correction is equal to that of with BSSE correction. For complexes **AIC**·*X*^−^ (*X* = Cl, Br, AcO, and H_2_PO_4_), the deviations of *R*_N–H_ and *R*_H_·*_X_* are within 0.004 and 0.15 Å, respectively. The corresponding deviations of angles *θ*_N–H_·*_X_* are within 6°. Therefore, BSSE correction is necessary for the optimization of this kind of system to some extent. Compared with the H–N bond in **AIC** (1.025 Å), obviously, the H–N bond is broken (0.447 Å elongation) and *R*_H···N_ = 1.4712 Å and *R*_H–F_ = 1.030 Å forming **AIC**^−^·HF. Namely, the pyrrole-H can be efficiently deprotonated by F^−^. In the cases of Cl^−^, Br^−^, AcO^−^, and H_2_PO_4_^−^, however, *R*_H–N_ values only change slightly (<0.044 Å elongation, see Table 1). This suggests that deprotonation indeed takes place from the acidic pyrrole-NH of the host chemosensor to form **AIC**^−^ and HF in the presence of F^−^. However, for Cl^−^, Br^−^, AcO^−^, and H_2_PO_4_^−^, the host chemosensor prefers to form the hydrogen-bonded complexes between **AIC** and *X*^−^ (*X* = Cl, Br, AcO, and H_2_PO_4_), rather than formation of the **AIC**^−^ form. Similar phenomena are also found for **1**–**5** (see Table SI, Supporting Information).

The interaction energies with BSSE corrections for complexes ***n***^−^·HF and ***n***·X^−^ (***n*** = **AIC** and **1**–**5**, *X* = Cl, Br, AcO, and H_2_PO_4_) are listed in Table 2. It is clear that the BSSE-corrected interaction energy (Δ*E*_BSSE_) of complex **AIC**^−^·HF is much more favorable for the distinct selectivity of F^−^ than other anions. The Δ*E*_BSSE_ of complex **AIC**^−^·HF is more than those of **AIC**·*X*^−^ (*X* = Cl, Br, AcO, and H_2_PO_4_) by about 20 kcal/mol. Therefore, the sensors can easily detect F^−^ in the presence of Cl^−^, Br^−^, AcO^−^, and H_2_PO_4_^−^. These calculation results are in good agreement with the reported experimental observations that intermolecular proton transfer (IPT) between chemosensor substrate **AIC** and F^−^ occurs when the concentration of F^−^ reaches a certain level with the addition of F^−^ to the sensor substrate solution [21]. Similar phenomena are also found for **1**–**5**. Thus, one can conclude that the host chemosensors have a much stronger affinity to F^−^ than to Cl^−^, Br^−^, AcO^−^, and H_2_PO_4_^−^ through intermolecular proton transfer, which leads to the formation of chemosensor anions by F^−^. The hydrogen bond acceptor F^−^ is stronger than those of Cl^−^, Br^−^, AcO^−^, and H_2_PO_4_^−^. On the other hand, the more pronounced the proton transfer reaction, the higher the intensity of the hydrogen bond interaction and the higher the stability of the complex [37,38]. Furthermore, the Δ*E*_BSSE_, *R*_H–N_, and *R*_H–_*_X_* (*X* = F, Cl, Br, AcO, and H_2_PO_4_) values of the complexes also support the distinct selectivity for F^−^ from Cl^−^, Br^−^, AcO^−^, and H_2_PO_4_^−^.

As the counterpoise correction was unreliable for the H-bond with fluorine in some cases [39], we calculated the interaction energies of **AIC**^−^·HF and **AIC**·*X*^−^ (*X* = Cl, Br, AcO, and H_2_PO_4_) at the MP2/6-31+G(d,p)//B3LYP/6-31+G(d,p) level. It turns out that the interaction energy of **AIC**^−^·HF (36.9 kcal/mol) is larger than that of Δ*E*_BSSE_ by 6.2 kcal/mol. The corresponding values of **AIC**·*X*^−^ (*X* = Cl, Br, AcO, and H_2_PO_4_) are larger than those of Δ*E*_BSSE_ about 8–10 kcal/mol. However, the interaction energy of **AIC**^−^·HF is larger than those of complexes **AIC**·*X*^−^(*X* = Cl, Br, AcO, and H_2_PO_4_) by about 10 kcal/mol at the MP2/6-31+G(d,p) level, respectively. It suggests that the interaction energy values of complexes at MP2/6-31+G(d,p) level support the distinct selectivity for F^−^ from Cl^−^, Br^−^, AcO^−^, and H_2_PO_4_^−^. Furthermore, we selected complexes **AIC**^−^·HF and **AIC**·*X*^−^ (*X* = Cl and Br) as representatives. The geometrical structures of these complexes were optimized using the MP2 method with 6-31+G(d,p) basis set. The interaction energies were calculated using the same methods as for the geometry optimizations and compared with the BSSE-corrected interaction energy (Δ*E*_BSSE_) at the B3LYP/6-31+G(d,p) level. The main geometrical parameters and the interaction energies of complexes **AIC**^−^·HF and **AIC**·*X*^−^ (*X* = Cl and Br) at the MP2/6-31+G(d,p) level are given in Table SII, Supporting Information. Comparing the results shown in Table SI with Table 1, one can find that the values of *R*_H···N_, *R*_H–F_, and angles *θ*_N–H_·_F_ of **AIC**^−^·HF at the MP2/6-31+G(d,p) level are similar to those of **AIC**^−^·HF with BSSE corrections at the B3LYP/6-31+G(d,p) level, the corresponding deviations are 0.004, 0.004 Å, and 4.0°, respectively. Similar phenomena are found for complexes **AIC**·*X*^−^ (*X* = Cl and Br). The value of interaction energy for complex **AIC**^−^·HF (37.2 kcal/mol) is larger than that of Δ*E*_BSSE_ by 6.5 kcal/mol. The corresponding interaction energies deviations for complexes **AIC**·*X*^−^ (*X* = Cl and Br) are larger than that of complex **AIC**^−^·HF. However, the interaction energy of **AIC**^−^·HF is larger than those of complexes **AIC**·*X*^−^ (*X* = Cl and Br) by 19 and 10 kcal/mol at the MP2/6-31+G(d,p) level, respectively. These results indicate that the *R*_H–N_, *R*_H–_*_X_* (*X* = F, Cl, and Br), and the interaction energy values of the complexes also support the distinct selectivity for F^−^ from Cl^−^ and Br^−^.

### 3.2. AIM and NBO Analysis

The AIM theory is often applied to study hydrogen bonds [40–42]. The characteristics of bond critical points (BCPs) are very useful to estimate the strength of hydrogen bonds. To gain a deeper insight into the fundamental nature of NH···*X* (*X* = F, Cl, Br, AcO, and H_2_PO_4_) hydrogen bonds, it is crucial to obtain reasonable estimates of their relative energies. In particular, the electron densities, *ρ*(r)*_bcp_*, and their Laplacians, ∇^2^*ρ*(r)*_bcp_*, evaluated at BCPs are frequently used as indicators of hydrogen bonds. More specifically, the kinetic *G*(r)*_bcp_* and potential *V*(r)*_bcp_* electron energy densities are often used to gain additional insight into the strength and nature of a given hydrogen bond. The local kinetic electron energy density can be evaluated from the values of *ρ*(r)*_bcp_* and ∇^2^*ρ*(r)*_bcp_* as [43]:

(1) $$G{\left(\text{r}\right)}_{bcp}=\frac{3}{10}{\left(3\pi \right)}^{2/3}\rho {\left(\text{r}\right)}_{bcp}^{5/3}+\frac{1}{6}\nabla^{2}\rho {\left(\text{r}\right)}_{bcp}$$

The kinetic energy density *G*(r)*_bcp_* is in turn related to the potential energy density *V*(r)*_bcp_* through the local statement of the virial theorem [44]:

(2) $$V{\left(\text{r}\right)}_{bcp}=\frac{\hbar^{2}}{4m}\nabla^{2}\rho {\left(\text{r}\right)}_{bcp}-2G{\left(\text{r}\right)}_{bcp}$$

The hydrogen bond energy *E*_HB_ (defined as −*D*_e_, where *D*_e_ is the hydrogen bond dissociation energy) in molecules can be estimated within the framework of the AIM analysis using the relationship [45]:

(3) $$E_{HB}=-D_{e}=0.5V{\left(\text{r}\right)}_{bcp}$$

The relationship (3) is used to estimate the energy of intermolecular hydrogen bonding. However, the values of the intramolecular hydrogen-bonding energy obtained by means of this equation are somewhat different from the real ones, particularly for intramolecular hydrogen transfer [46]. One of the most reliable approaches to estimating the energy of intramolecular hydrogen bonding is the conformational method. Unfortunately, such a scheme cannot be applied in our calculations because of the lack of conformer. The second one by Musin and Mariam [47] is disposed to correlate *E*_HB_ with the interatomic separation (in Å) from donor (D) to acceptor (A) according to the empirical relationship. However, this relationship does not suit the hydrogen bonds in the chemosensors under investigation. Because the pyrrole-NH of the chemosensors are deprotonated by F^−^ and form complexes ***n***^−^·HF (***n*** = **AIC** and **1**–**5**), the hydrogen bonds in complexes ***n***^−^·HF can be regarded as intermolecular hydrogen bonds between chemosensors anions and HF. Therefore, the *E*_HB_ are estimated by this relationship within the framework of the AIM analysis.

The *ρ*(r)*_bcp_*, ∇^2^*ρ*(r)*_bcp_*, and *E*_HB_ of the complexes **AIC**·HF^−^ and **AIC**·*X*^−^ (*X* = Cl, Br, AcO, and H_2_PO_4_) are given in Table 3. The corresponding topological parameters of the complexes ***n***^−^·HF and ***n***·*X*^−^ (***n*** = **1**–**5**, *X* = Cl, Br, AcO, and H_2_PO_4_) are given in Table SIII, Supporting Information. As a general rule, hydrogen bonds are characterized by positive values of ∇^2^*ρ*(r)*_bcp_* and low *ρ*(r)*_bcp_* values. Covalent bonds (shared interactions) have negative ∇^2^*ρ*(r)*_bcp_* values and high values of *ρ*(r)*_bcp_*, whereas the values of ∇^2^*ρ*(r)*_bcp_* become positive when the bonds are of a ionic nature [48]. The results displayed in Tables 3 and SIII reveal that the *ρ*(r)*_bcp_* and ∇^2^*ρ*(r)*_bcp_* values of H···N in ***n***^−^·HF (***n*** = **AIC** and **1**–**5**) are about 0.09 and 0.06 au, respectively. This suggests that the interactions between N and H in ***n***^−^·HF (***n*** = **AIC** and **1**–**5**) are hydrogen bonds in nature. The *ρ*(r)*_bcp_* and ∇^2^*ρ*(r)*_bcp_* values of H–F in ***n***^−^·HF (***n*** = **AIC** and **1**–**5**) are about 0.25 and −1.1 au, respectively. Hence, H–F bonds of ***n***^−^·HF (***n*** = **AIC** and **1**–**5**) are of a covalent bond nature. On the contrary, for complexes ***n***·*X*^−^ (***n*** = **AIC** and **1**–**5**, *X* = Cl, Br, AcO, and H_2_PO_4_), BCPs at H–N provides ∇^2^*ρ*(r)*_bcp_* < 0 (about −1.5 au) and high positive values for *ρ*(r)*_bcp_* (about 0.3 au), which are characteristics of covalent type interactions. For H···X bonds in complexes ***n***·*X*^−^ (***n*** = **1**–**5**, *X* = Cl, Br, AcO, and H_2_PO_4_), the values of *ρ*(r)*_bcp_* and ∇^2^*ρ*(r)*_bcp_* are about 0.04 and 0.06 au, respectively. This indicates that H···*X* bonds in complexes ***n***·*X*^−^ (***n*** = **1**–**5**, *X* = Cl, Br, AcO, and H_2_PO_4_) are of hydrogen bond nature. Furthermore, the *E*_HB_ values of H···N and H···X in complexes confirm the expectation. The *E*_HB_ values of H···N bonds in ***n***^−^·HF (***n*** = **AIC** and **1**–**5**) are about 27 kcal/mol, while the H···X bonds in ***n***·*X*^−^ (***n*** = **AIC** and **1**–**5**, *X* = Cl, Br, AcO, and H_2_PO_4_) are 4–20 kcal/mol. The above results show qualitative agreement with the results based on their geometries.

It is well known that the electronic reorganization derived from the formation of a hydrogen bond is associated with a charge transfer between the two moieties of the complex [49]. The overall NBO charge transfer has been evaluated by summing up the NBO atomic charges on the two moieties of each hydrogen-bonded complex (the host chemosensor and halides). We select the parent compound **AIC** as representative of the system under investigation. The calculated NBO charge densities for **AIC**^−^·HF and **AIC**·*X*^−^ (*X* = Cl, Br, AcO, and H_2_PO_4_) are collected in Table SIV in supplementary information. It clearly shows that the sum of charges on the **AIC**^−^ moiety is −0.856, while the corresponding value of HF is only −0.144 in complex **AIC**^−^·HF. The sum of charges on the **AIC** moiety and Cl^−^ in **AIC**·Cl^−^ are −0.098 and −0.902, respectively. The corresponding values in **AIC**·Br^−^, **AIC**·AcO^−^, and **AIC**·H_2_PO_4_^−^ are −0.072 and −0.928, −0.120 and −0.88, and −0.060 and −0.933, respectively. Hence, the NBO charge analysis also indicates that the proton is almost completely abstracted by F^−^. In the cases of Cl^−^, Br^−^, AcO^−^, and H_2_PO_4_^−^, however, the host chemosensor prefers to form the hydrogen-bonded complexes between **AIC** and Cl^−^, Br^−^, AcO^−^, and H_2_PO_4_^−^, rather than the **AIC**^−^ form.

### 3.2. Electronic Properties

**Figure 1:**
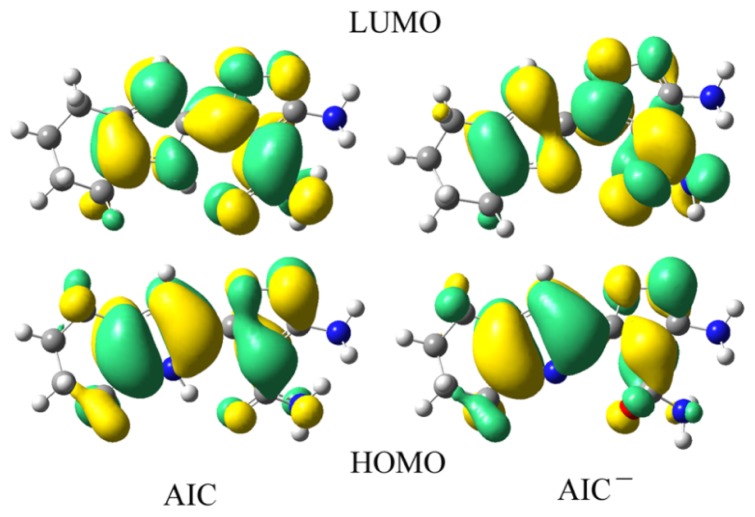
Frontier molecular orbital (FMOs) of **AIC** and **AIC**^−^ in S_0_ at the B3LYP/6- 31+G(d,p) level.

It is useful to examine the frontier molecular orbitals (FMOs) of the compounds under investigation. The origin of the geometric difference introduced by excitation can be explained, at least in qualitative terms, by analyzing the change in the bonding character of the orbitals involved in the electronic transition for each pair of bonded atoms. An electronic excitation results in some electron density redistribution that affects the molecular geometry [49,50]. The qualitative molecular orbital representations of FMOs for **AIC** and **AIC**^−^ in S_0_ are shown in Figure 1. The corresponding qualitative molecular orbital representations of FMOs for **AIC** and **AIC**^−^ in S_1_ are shown in Figure SI, Supporting Information. The major assignments of the lowest electronic transitions for **AIC** and **AIC**^−^ are mainly as HOMO→LUMO, which corresponds to a π–π^*^ excited singlet state. From Figure 1, one can see that both the HOMO and LUMO of **AIC** and **AIC**^−^ are spread over the whole conjugated molecule. A careful inspection of the results displayed in Figure 1 reveals that the vertical S_0_→S_1_ transition of **AIC** and **AIC**^−^ shows the intramolecular charge transfer (ICT) in nature. Analysis of the FMOs for **AIC** and **AIC**^−^ indicates that the excitation of the electron from the HOMO to LUMO makes the electronic density flow mainly from the 4,5,6,7-tetrahydro-1*H*-indole moiety (part A) to 3-aminoisoxazole-4-carboxamide moiety (part B). The HOMO of **AIC** has contributions of 69.2% and 30.8% on parts A and B, while the corresponding values of LUMO are 35.5% and 64.5%, respectively. The HOMO of **AIC**^−^ has contributions of 68.9% and 31.1% on parts A and B, while the corresponding values of LUMO are 29.0% and 71.0%, respectively. Hence, the percentage of charge transfer from Parts A to B is 38.9% in **AIC**^−^, which is greater than that of 5.2% in **AIC**. It is obvious that deprotonation strengthens the electron-donating ability of part A, suggesting a stronger coupling between parts A and B. The molecular p-conjugation in **AIC**^−^ becomes higher than that of **AIC**. As a consequence, the electron can move more fluently from parts A to B. This suggests that the charge-transfer character of **AIC**^−^ is stronger than that of **AIC**. The ICT transition in the chemosensor system becomes much easier after deprotonation, resulting in a red shift between their UV-vis spectra. Similar phenomena are also found for S_1_ (see Figure SI). However, the intensity of fluorescence may be weak because the **AIC**^−^ has a worse conjugation due to large twist angles between Parts A and B. The dihedral values of between Parts A and B for **AIC** and **AIC**^−^ are 179.0 and 78.7° respectively, suggesting that the molecular p-conjugation in **AIC**^−^ becomes lower than that of **AIC**. These results reveal that the deprotonation by F^−^ has obvious effects on the distribution of FMOs, resulting in a red shift between their fluorescence spectra and decreasing the intensity of fluorescence.

### 3.3. Optical Properties

The original color and emissions change upon addition of F^−^ because of the formation of anions. The addition of F^−^ leads to an intermolecular proton transfer (IPT) between chemosensor **AIC** and F^−^ which forms the deprotonated anion **AIC**^−^ and HF. This shifts the equilibrium from **AIC** towards **AIC**^−^ (see Scheme I). Hence, the absorption and emission of **AIC** (340 and 340 nm) are decreased and a new absorption and emission of **AIC**^−^ has been observed when adding enough amounts of fluoride to the sensor’s solution. In contrast, the addition of Cl^−^, Br^−^, AcO^−^, and H_2_PO_4_^−^ leaves its absorption and emission spectra almost unchanged. Therefore, the absorption and emission bands can be assigned to **AIC** and **AIC**^−^ before and after the addition of F^−^, respectively. The driving force for the changing of the color and emission is the intermolecular proton transfer. The bathochromic or hypsochromic shifts between the two characteristic absorptions and emissions of **AIC** and **AIC**^−^ are chosen to calculate the colorimetric and ratiometric fluorescent fluoride anion chemosensor.

Table 4 presents the absorption *λ*_abs_ wavelengths, assignments, and the oscillator strength *f* for **AIC** and **1**–**5** and their anions in CH_3_CN at the TD-B3LYP/6-31+G(d,p) level. The predicted *λ*_abs_ values are in excellent agreement with experimental results [21]. Namely, *λ*_abs_ = 340 and 375 nm before and after the addition of F^−^. The bathochromic shift between the two characteristic *λ*_abs_ values for **AIC** and **AIC**^−^ is 34 nm, which is comparable to the experimental 35 nm. The values of oscillator strength *f* for **AIC** and **AIC**^−^ are 0.67 and 0.55, respectively. This indicates that the intensity absorbance of **AIC**^−^ is weaker than that of **AIC** after the addition of F^−^. However, with increasing F^−^ concentration, the intensity of the absorbance for **AIC** at 340 nm decreases significantly and the intensity of a new absorption peak for **AIC**^−^ increases because the formation of **AIC**^−^ shifts the equilibrium from **AIC** towards **AIC**^−^. Furthermore, the higher molecular p-conjugation in **AIC**^−^ enhances the intensity of absorption for **AIC**^−^. Thus, this result is found thanks to the computational approach, so appropriate electronic transition energies can be predicted at these levels for this kind of chemosensor. The successful simulations indicate that the observed colorimetric and fluorescent signals truly originate from the formation of **AIC**^−^ anion.

In Table 4, one can see that the *λ*_abs_ of **1** shows a slightly hypsochromic shift, while **2**, **3**, and **5** have slight bathochromic shifts compared with that of the parent compound **AIC**, with the maximum deviation being less than 38 nm. However, **4** has a strong bathochromic shift compared with that of the parent compound **AIC**, the deviation being 348 nm. In general, a larger oscillator strength corresponds to a larger experimental absorption coefficient or stronger fluorescence intensity. The *f* values of **2** and **5** are larger, while the corresponding *f* values of **1**, **3**, and **4** are smaller than that of **AIC**, particularly for **4**. This suggests that **2** and **5** correspond to more intensive spectra. For ***n***^−^ (***n*** = **1**–**5**) of the substituted derivatives, it is found that the *λ*_abs_ of **1**^−^ and **2**^−^ show slightly hypsochromic shifts, while **3**^−^ and **5**^−^ have slight bathochromic shifts compared with that of **AIC**^−^, the deviations are within 29 nm. However, **4**^−^ has a strong bathochromic shift compared with that of the parent compound **AIC** (the deviation being 82 nm). The *f* values of **1**^−^–**5**^−^ are larger than that of **AIC**^−^ except that the value of *f* for **4** is less than that for **AIC**^−^. This clearly shows that the functional group in **4** has more influence on the shifts of *λ*_abs_ for the substituted derivative and its anion, while the functional groups in **1**, **2**, **3**, and **5** do not significantly affect the *λ*_abs_ of the substituted derivatives and their anions. Furthermore, derivatives **1**–**5** and their anions show a more intensive spectrum than those of **AIC** and its anion except for **4**.

On the basis of the results displayed in Table 4 and taking into account the Δ*E*_BSSE_, one can conclude that the substituted derivatives **1**–**4** are expected to be fluoride chemosensors. The bathochromic or hypsochromic shifts between the two characteristic *λ*_abs_ values of ***n*** and ***n***^−^ (***n*** = **1**–**4**, *i.e.*, *λ*_abs_ values before and after the addition of F^−^) are 12, 9, 20, and 232 nm, respectively. Therefore, **1**–**5** show changes in their UV-vis absorption spectra upon the addition of F^−^, resulting in high selectivity for fluoride detection over other anions, such as Cl^−^, Br^−^, AcO^−^, and H_2_PO_4_ in CH_3_CN. However, **5** does not seem to be a chromogenic chemosensor because the shift between the two characteristic *λ*_abs_ values of **5** and **5**^−^ is only 4 nm and the original color of the solution of **5** has no obvious change to the naked eye upon addition of F^−^.

Table 5 presents the fluorescence *λ*_fl_ wavelengths, assignments, and the oscillator strength *f* for **AIC** and **1**–**5** and their anions in CH_3_CN at the TD-BLYP/6-31+G(d,p) level. The predicted *λ*_fl_ values are in agreement with experimental results [21]. Namely, *λ*_fl_ = 400 and 432 nm before and after the addition of F^−^, respectively. The bathochromic shift between the two characteristic *λ*_fl_ values for **AIC** and **AIC**^−^ is 56 nm, which is comparable to the experimental 32 nm. From Table 5, one can deduct that the *λ*_fl_ values of **2** and **3** have slight bathochromic shifts, while the corresponding values of **1**, **4**, and **5** show strong hypsochromic shifts compared with that of **AIC**. The *f* values of **2** and **5** are larger while the *f* values of **1** and **3** are smaller than that of **AIC**. The *f* value of **4** is the smallest among the substituted derivatives. For ***n***^−^ (***n*** = **1**–**5**) of the substituted derivatives, the *λ*_fl_ values of **1**^−^and **5**^−^ have hypsochromic shifts, while the corresponding values of **2**^−^–**4**^−^ show more bathochromic shifts compared with that of **AIC**^−^. Furthermore, the *f* values of **AIC**^−^ and **2**^−^–**4**^−^ are small (<0.1), indicating that the intensity of fluorescence for **AIC**^−^ and **2**^−^–**4**^−^ is weak. The results displayed in Table 5 and the Δ*E*_BSSE_ values indicate that all the substituted derivatives ***n*** (***n*** = **1**–**5**) are expected to be promising candidates for fluorescent fluoride chemosensors. The bathochromic or hypsochromic shifts between the two characteristic *λ*_fl_ values of ***n*** and ***n***^−^ (*i.e.*, *λ*_fl_ values before and after the addition of F^−^) are 62, 308, 111, 65, and 24 nm, respectively. Therefore, the original emissions of **1**–**5** may be quenched and new weak emissions appear upon the addition of F^−^.

## 4. Conclusions

Our calculated results for both the host-guest interaction and the nature of UV-vis and fluorescent signaling for **AIC** in the presence of F^−^ are in good agreement with the reported experimental observations. The host chemosensor **AIC** has a much stronger affinity to F^−^ than to Cl^−^, Br^−^, AcO^−^, and H_2_PO_4_^−^ through intermolecular proton transfer, which leads to the formation of chemosensor anions by F^−^. The AIM theory and NBO charge analysis of the complexes consisting of **AIC** and *X*^−^ (*X* = F, Cl, Br, AcO, and H_2_PO_4_) confirm that the protons are almost completely abstracted by F^−^. FMOs analyses have shown that the vertical electronic transitions of absorption and emission for **AIC** and **AIC**^−^ forms corresponding to the sensing signals are characterized as intramolecular charge transfer (ICT). The ICT transition becomes much more efficient when the chemosensor is deprotonated by F^−^. However, the intensity of fluorescence of **AIC**^−^ is weak because the **AIC**^−^ has a worse conjugation due to large twisted angles between 4,5,6,7-tetrahydro-1*H*-indol moiety and the 3-aminoisoxazole-4-carboxamide moiety. The study of substituent effects suggests that all the substituted derivatives are expected to be promising candidates for fluoride chemosensors both in UV-vis and fluorescence spectra except for the substituted derivative with benzo[*d*]thieno[3,2-*b*]thiophene fragment that can serve as ratiometric fluorescent fluoride chemosensor only.

## Supplementary Materials



## Figures and Tables

**Scheme I:**
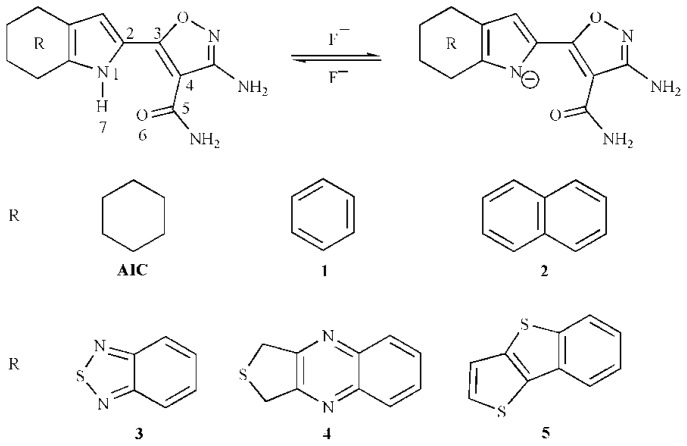
Geometries of 3-amino-5-(4,5,6,7-tetrahydro-1*H*-indol-2-yl)isoxazole-4- carboxamide (**AIC**) and its derivatives, along with atom numbering.

**Table 1:** The distances of *R*_N–H_ and *R*_H_·*_X_* (in angstroms) and angles of *θ*_O–H_·*_X_* (in deg) with and without basis set superposition error (BSSE) corrections (in kcal/mol) for complexes **AIC**^−^·HF and **AIC**·*X*^−^ (*X* = Cl, Br, AcO, and H_2_PO_4_) at the B3LYP/6-31+G(d,p) level.

	without BSSE			with BSSE		
		
Complexes	*R*_H7···N1_	*R*_H7–_*_X_*	*θ*_N1···H7–_*_X_*	*R*_H7···N1_	*R*_H7–_*_X_*	*θ*_N1···H7–_*_X_*
**AIC**	1.025					
**AIC**^−^·HF	1.467	1.031	170.8	1.472	1.030	170.8
**AIC**·Cl^−^	1.046	2.126	156.6	1.045	2.139	156.1
**AIC**·Br^−^	1.036	2.320	153.2	1.032	2.479	147.4
**AIC**·AcO^−^	1.069	1.614	170.7	1.067	1.630	169.5
**AIC**·H_2_PO_4_^−^	1.049	1.671	176.1	1.048	1.686	175.3

**Table 2:** The interaction energies with BSSE corrections Δ*E*_BSSE_ (in kcal/mol) of complexes **n**^−^·HF, **n**·Cl^−^, **n**·Br^−^, **n**·AcO^−^, and **n**·H_2_PO_4_^−^ (**n** = **AIC** and **1**–**5**) at the B3LYP/6-31+G(d,p) level.

*n*	*n*^−^·HF	*n*·Cl^−^	*n*·Br^−^	*n*·AcO^−^	*n*·H^2^PO_4_^−^
AIC	−30.7	−6.9	−4.1	−12.0	−16.4
1	−34.9	−9.4	−6.3	−14.6	−13.5
2	−37.4	−11.2	−8.0	−16.4	−14.7
3	−41.9	−14.3	−7.3	−19.1	−23.6
4	−42.2	−14.2	−6.9	−18.4	−16.5
5	−39.1	−11.9	−8.6	−17.9	−23.4

**Table 3:** Electronic density at bond critical points (BCP) *ρ*(r)*_bcp_*, the Laplacian ∇^2^*ρ*(r)*_bcp_* (all in au), and the bond energy *E*_HB_ (in kcal/mol) of complexes **AIC**^−^·HF and **AIC**·*X*^−^ (*X* = Cl, Br, AcO, and H_2_PO_4_) at the B3LYP/6-31+G(d,p) level.

	H_7_···N_1_			H_7_–*X*		
		
*X*	*ρ*(r)*_bcp_*	∇_2_*ρ*(r)*_bcp_*	*E*_HB_	*ρ*(r)*_bcp_*	∇_2_*ρ*(r)*_bcp_*	*E*_HB_
F	0.0926	0.0429	−29.6	0.2506	−1.026	−145.9
Cl	0.3080	−1.6563	−156.8	0.0302	0.0609	−6.1
Br	0.3206	−1.7499	−163.1	0.0183	0.040968	−3.1
AcO	0.2887	−1.5106	−146.7	0.0545	0.138816	−13.1
H_2_PO_4_	0.3057	−1.6347	−155.9	0.0459	0.131748	−10.9

**Table 4:** Absorption (*λ*_abs_) wavelengths (in nm), the oscillator strength *f*, and assignments (coefficient) for **AIC** and **1**–**5** and their anions in CH_3_CN at the TD-BLYP/6-31+G(d,p) level, along with available experimental data.

	Neutral				Anion			
		
Compounds	λ_abs_	*f*	Assignments	Exp *	λ_abs_	*f*	Assignments	Exp *
AIC	341	0.67	H→L (0.70)	340	375	0.55	H→L (0.70)	375
1	334	0.45	H-1→L (0.69)		346	0.64	H-1→L (0.69)	
2	357	0.80	H-1→L (0.69)		366	0.80	H-1→L (0.68)	
3	368	0.53	H-1→L (0.62)		388	0.80	H-1→L (0.66) H→L+1 (0.23)	
4	689	0.07	H→L (0.71)		457	0.21	H-1→L (0.54) H-2→L (0.38)	
5	379	0.93	H-1→L (0.69)		383	0.93	H-1→L (0.69) H→L + 1 (0.13)	

* Experimental results were taken from [21].

**Table 5:** Fluorescence (*λ*_fl_) wavelengths (in nm), the oscillator strength *f*, and assignments (coefficient) for **AIC** and **1**–**5** and their anions in CH_3_CN at the TD-BLYP/6-31+G(d,p) level, along with available experimental data.

	neutral				anion			
		
Compounds	λ_fl_	*f*	Assignments	Exp*	λ_fl_	*f*	Assignments	Exp*
AIC	409	0.42	H←L (0.68) H-1←L (0.10)	400	465	0.02	H←L + 1 (0.75)	432
1	471	0.27	H←L (0.68) H-1←L (0.16)		409	0.48	H-1←L (0.69)	
2	413	0.65	H←L (0.12) H-1←L (0.65)		721	0.04	H←L (0.70)	
3	416	0.37	H←L + 1 (0.28) H-1←L (0.48)		527	0.07	H←L + 1 (0.69)	
4	579	0.02	H←L + 1 (0.45) H-1←L (0.53)		514	0.25	H←L + 1 (0.29) H-1←L (0.60)	
5	462	0.63	H-1←L (0.66) H←L (0.13)		438	0.85	H←L + 1 (0.36) H-1←L (0.59)	

* Experimental results were taken from [21].
