# Supplementary material for: A DFT Study of Pyrrole-Isoxazole Derivatives as Chemosensors for Fluoride Anion
Source: Int J Mol Sci. 2012 Sep 5;13(9):10986–99. doi: 10.3390/ijms130910986 (PMC3472725; doi:10.3390/ijms130910986)
Supplement: Supplementary file 1 [file ijms-13-10986-s001.pdf]

Supplementary Information

Table SI. Main geomccccetrical parameters (bond lengths and angles in angstroms and degrees, respectively) for complexes $\boldsymbol{n}^{-}$·HF and $\boldsymbol{n}\cdot X^{-}$ ($\boldsymbol{n}$ = 1–5, X = Cl, Br, AcO, and H_2PO_4) at the B3LYP/6-31G(d,p) level.

	n^{-}·HF			n·Cl^{-}			n·Br^{-}		
n	$R_{N\cdots H}$	R_{H-F}	$\theta_{N\cdots H-F}$	R_{N-H}	$R_{H\cdots Cl}$	$\theta_{N-H\cdots Cl}$	R_{N-H}	$R_{H\cdots Br}$	$\theta_{N-H\cdots Br}$
1	1.474	1.027	174.5	1.053	2.055	170.2	1.041	2.302	167.9
2	1.479	1.024	175.5	1.054	2.050	170.0	1.041	2.311	167.4
3	1.490	1.019	176.0	1.055	2.042	168.6	1.039	2.235	174.5
4	1.489	1.015	176.0	1.055	2.049	167.4	1.040	2.231	176.0
5	1.489	1.020	174.6	1.058	2.019	171.0	1.046	2.256	169.1

	n·AcO^{-}			n·$H_2PO_4^{-}$		
n	R_{N-H}	$R_{H\cdots O}$	$\theta_{N-H\cdots O}$	R_{N-H}	$R_{H\cdots OI}$	$\theta_{N-H\cdots O}$
1	1.077	1.582	172.1	1.061	1.613	171.6
2	1.080	1.567	172.6	1.062	1.601	172.8
3	1.090	1.534	171.6	1.046	1.720	170.4
4	1.089	1.555	169.7	1.070	1.570	172.6
5	1.084	1.555	172.8	1.042	1.699	172.9

Table SII. Main geometrical parameters (bond lengths and angles in angstroms and degrees, respectively) and the interaction energies of complexes **AIC**$^{-}$·HF and **AIC**·X^{-} (X = Cl and Br) at the MP2/6-31+G(d,p) level.

Complexes	$R_{H7\cdots N1}$	R_{H7-X}	$\theta_{N1\cdots H7-X}$	ΔE
**AIC**	1.022			
**AIC**$^{-}$·HF	1.468	1.026	174.8	−37.2
**AIC**·Cl^{-}	1.040	2.138	165.4	−18.1
**AIC**·Br^{-}	1.035	2.254	171.4	−27.3

Table SIII. Electronic density at BCP $\rho(\mathrm{r})_{\mathrm{bcp}}$, the Laplacian $\nabla^2\rho(\mathrm{r})_{\mathrm{bcp}}$ (all in au), and the bond energy E_{HB} (in kcal/mol) of complexes $\boldsymbol{n}$·HF^{-} and $\boldsymbol{n}\cdot X^{-}$ ($\boldsymbol{n}$ = 1–5, X = Cl, Br, AcO, and H_2PO_4) at the B3LYP/6-31G(d,p) level.

	n^{-}·HF						n·Cl^{-}					
	H···N			H–F			H–N			H···Cl		
n	$\rho(\mathrm{r})_{bcp}$	$\nabla^2\rho(\mathrm{r})_{bcp}$	E_{HB}	$\rho(\mathrm{r})_{bcp}$	$\nabla^2\rho(\mathrm{r})_{bcp}$	E_{HB}	$\rho(\mathrm{r})_{bcp}$	$\nabla^2\rho(\mathrm{r})_{bcp}$	E_{HB}	$\rho(\mathrm{r})_{bcp}$	$\nabla^2\rho(\mathrm{r})_{bcp}$	E_{HB}
**1**	0.0918	0.0472	−29.3	0.2529	−1.055	−148.4	0.3002	−1.5895	−152.8	0.0361	0.0655	−7.4
**2**	0.0905	0.0529	−28.7	0.2554	−1.0875	−150.9	0.2994	−1.5825	−152.5	0.0365	0.0659	−7.5
**3**	0.0876	0.0633	−27.2	0.2698	−1.1458	−155.1	0.2977	−1.5692	−151.6	0.0373	0.0663	−7.6
**4**	0.0855	0.0703	−26.2	0.2628	−1.1893	−158.2	0.2980	−1.5725	−151.7	0.0367	0.0656	−7.5
**5**	0.0889	0.0594	−27.9	0.2587	−1.1280	−154.1	0.2960	−1.5560	−150.7	0.0391	0.0678	−8.1

Table SIII. *Cont.*

	$n^{-}\cdot Br^{-}$						$n\cdot H_2PO_4^{-}$					
	H–N			H···Br			H–N			H···H_2PO_4		
n	$\rho(r)_{bcp}$	$\nabla^2\rho(r)_{bcp}$	E_{HB}	$\rho(r)_{bcp}$	$\nabla^2\rho(r)_{bcp}$	E_{HB}	$\rho(r)_{bcp}$	$\nabla^2\rho(r)_{bcp}$	E_{HB}	$\rho(r)_{bcp}$	$\nabla^2\rho(r)_{bcp}$	E_{HB}
1	0.3108	−1.6676	−158.2	no*	no	no	0.2927	−1.5396	−149.1	0.0545	0.1507	−13.6
2	0.3111	−1.6688	−158.4	0.0255	0.0474	−4.4	0.2908	−1.5227	−148.2	0.0562	0.1532	−14.3
3	0.3125	−1.6603	−159.9	0.0301	0.0536	−5.5	0.3060	−1.6466	−155.8	0.0409	0.1222	−9.4
4	0.3116	−1.6542	−159.4	0.0304	0.0537	−5.6	0.2840	−1.4675	−144.7	0.0613	0.1576	−16.2
5	0.3072	−1.6392	−156.3	0.0286	0.0504	−5.1	0.3097	−1.6682	−158.0	0.0422	0.1321	−9.9
	$n^{-}\cdot AcO^{-}$											
	H–N			H···AcO								
n	$\rho(r)_{bcp}$	$\nabla^2\rho(r)_{bcp}$	E_{HB}	$\rho(r)_{bcp}$	$\nabla^2\rho(r)_{bcp}$	E_{HB}						
1	0.2794	−1.4316	−142.0	0.0622	0.1455	−15.9						
2	0.2764	−1.4055	−140.5	0.0647	0.1466	−16.9						
3	0.2685	−1.3389	−136.4	0.0710	0.1462	−19.5						
4	0.2637	−1.2993	−133.9	0.0747	0.1441	−21.2						
5	0.2737	−1.3845	−139.1	0.0669	0.1471	−17.8						

Table SIV. The NBO charges of complexes **AIC**$^{-}$·HF and **AIC**·X^{-} (X = Cl, Br, AcO, and H_2PO_4).

Complexes	X^{-}	AIC	AIC^{-}	HX
**AIC**$^{-}$·HF			−0.8564	−0.1436
**AIC**·Cl^{-}	−0.9015	−0.0981		
**AIC**·Br^{-}	−0.9280	−0.0717		
**AIC**·AcO^{-}	−0.8801	−0.1199		
**AIC**·$H_2PO_4^{-}$	−0.9334	−0.0596		

Figure SI. FMOs of **AIC** and **AIC**$^{-}$ in S_1 at the TD-CAM-B3LYP/6-31+G(d,p) level.

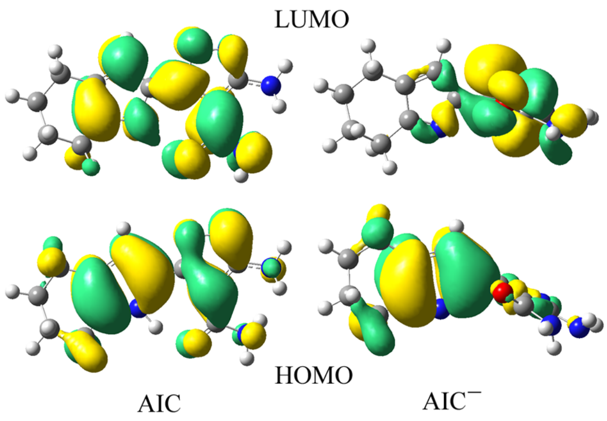

© 2012 by the authors; licensee MDPI, Basel, Switzerland. This article is an open access article distributed under the terms and conditions of the Creative Commons Attribution license (http://creativecommons.org/licenses/by/3.0/).
